# Exercise capacity, respiratory mechanics and posture in mouth breathers

**DOI:** 10.1590/S1808-86942011000500020

**Published:** 2015-10-22

**Authors:** Renata Tiemi Okuro, André Moreno Morcillo, Eulália Sakano, Camila Isabel Santos Schivinski, Maria Ângela Gonçalves Oliveira Ribeiro, José Dirceu Ribeiro

**Affiliations:** 1 Master's degree student in child and adolescent health, UNICAMP. Physical therapist; 2 Associate professor of pediatrics, Campinas State University. Associate professor of the Pediatrics Department, Medical School, Campinas State University; 3 Doctoral degree in otorhinolaryngology, Campinas State University. Assistant professor of the Otorhinolaryngology Department, Medical School, UNICAMP; 4 Doctoral degree in child and adolescent health, UNICAMP. Assistant professor at the Santa Catarina State University; 5 Doctoral degree in child and adolescent health, UNICAMP. Coordinator of the Laboratory of Lung Physiology of the Center for Pediatric Investigation, Medical School, UNICAMP; 6 Associate professor in pediatrics, Campinas State University. Associate professor of the Pediatrics Department, Medical School, Campinas State University

**Keywords:** exercise tolerance, mouth breathing, posture, respiratory mechanics

## Abstract

**Abstract:**

Chronic and persistent mouth or oral breathing (OB) has been associated with postural changes. Although posture changes in OB causes decreased respiratory muscle strength, reduced chest expansion and impaired pulmonary ventilation with consequences in the exercise capacity, few studies have verified all these assumptions.

**Objective:**

To evaluate exercise tolerance, respiratory muscle strength and body posture in oral breathing (OB) compared with nasal breathing (NB) children.

**Material and method:**

A cross-sectional contemporary cohort study that included OB and NB children aged 8-11 years old. Children with obesity, asthma, chronic respiratory diseases, neurological and orthopedic disorders, and cardiac conditions were excluded. All participants underwent a postural assessment, maximal inspiratory pressure (MIP), maximal expiratory pressure (MEP), the six-minute walk test (6MWT), and otorhinolaryngologic evaluation.

**Results:**

There were 107 children (45 OB and 62 NB). There was an association between abnormal cervical posture and breathing pattern: 36 (80.0%) OB and 30 (48.4%) NB presented abnormal head posture (OR=4.27 [95% CI: 1.63-11,42], *p*<0.001). The mean MIP and MEP were lower in OB (*p*=0.003 and *p*=0.004).

**Conclusion:**

OB children had cervical spine postural changes and decreased respiratory muscle strength compared with NB.

## INTRODUCTION

The mouth breathing syndrome may be characterized by mixed or mouth supplementary breathing replacing an exclusively nasal breathing pattern. This syndrome presents functional, structural, postural, biomechanical, occlusal, and behavioral involvement^1^,^2^.

An altered respiration pattern in the mouth breathing syndrome implies necessary adaptive body postures.^3^ Such individuals anteriorize their heads and extend their necks to facilitate air flow through the mouth; more air passes through the pharynx, which reduces airway resistance^4^,^5^. This adaptation results in muscle unbalance and alters the postural axis, thereby disorganizing the muscle groups. The diaphragm and abdominal muscles are less active and become less synergic^6^.

Oral breathing may also inhibit nasal afferent nerves (trigeminal autonomic and sympathetic nerve), which regulate depth of breathing and airway caliber. Nasal block increases resistance and decreases lung compliance, thereby restraining thoracic expansion and alveolar ventilation^7^.

It is thought that a disorganized posture, starting in the neck, reduces diaphragmatic work, which in turn decreases thoracic expansion. These adaptations interfere with pulmonary ventilation and exercise capacity^8^,^9^. These changes in mouth breathing children have not been investigated in depth, in the literature.

It is important to learn and identify the effects of the mouth breathing syndrome on lung function and other systems, as an early diagnosis makes it possible to intervene earlier and more effectively to avoid additional involvement.

The purpose of this study was to assess tolerance to submaximal exercise, respiratory muscle strength, and postural pattern in mouth breathing and nose breathing children.

## METHOD

**Figure 1:**
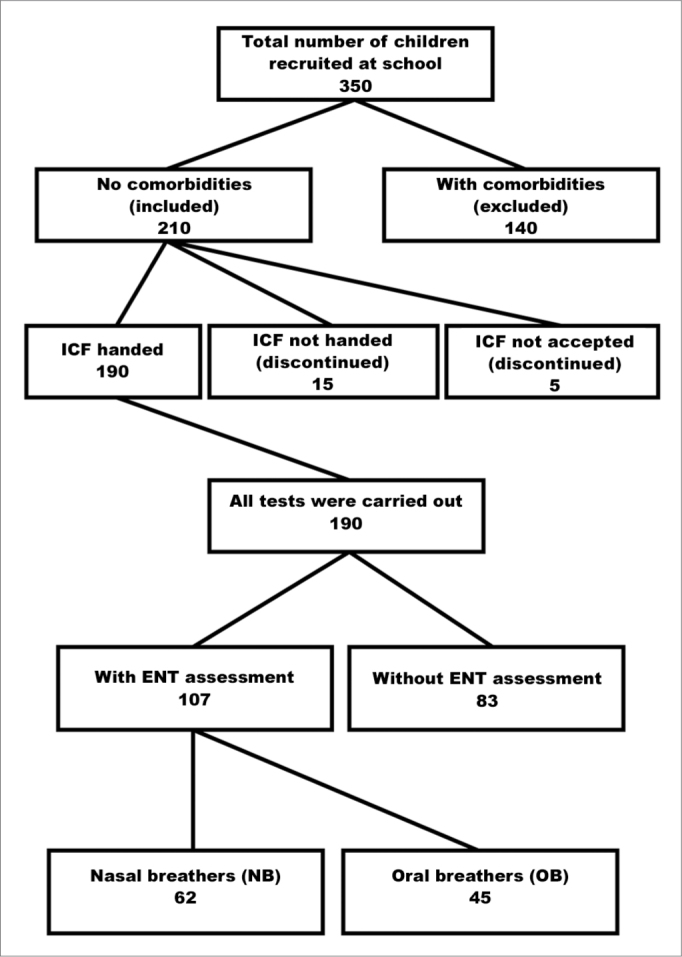
Sample screening. FICF (TCLE) - Free informed consent form.

The sample comprised all children aged 8 to 11 years enrolled in the morning study period at the Basic Education D. Ana José Bodini Januario School in Hortolandia, which is located in metropolitan Campinas (Sao Paulo State). This age range was chosen because of ease of testing, and because it included children in 1^st^ to 4^th^ grade of basic education (Figure 1).

Inclusion criteria: mouth or nose breathing children without the comorbidities listed below as exclusion criteria.

Exclusion criteria: body mass index above the 95^th^ percentile, asthma, chronic respiratory diseases, neurological or orthopedic conditions, heart diseases, and having undergone tonsillectomy and adenoidectomy.

The medical team of the Otorhinolaryngology Department of the Clinic Hospital (UNICAMP) evaluated and classified the subjects in two groups: mouth or oral breathers (OB) and nose breathers (NB). An otorhinolaryngological examination and a questionnaire for parents or caretakers were the basis of the diagnosis. The questionnaire contained questions on the health history of children and focused on the type of breathing (mouth or nose), its onset, and the presence of rhinitis (Figure 1).

Posture was assessed using the New York test; the respiratory muscle strength was assessed using the maximal inspiratory pressure (MIP) and the maximal expiratory pressure (MEP). Next, children performed the six-minute walking test (6MWT). Trained physical therapists carried out these assessments; the same professional performed the each test, and was not aware of the results of the other tests.

The New York test is an objective method for assessing posture in thirteen body segments^10^. It has a scoring system that provides a quantitative analysis to classify postural disorders. Posture may be classified as severe, moderate, and normal^11^. At the end of the test, subjects were classified as having a normal or altered general posture, and a normal or altered neck posture.

MIP and MEP measurements were made using a manovacuometer (MV-120, Ger-Ar-SP Com. Equip. Ltda.) with a tracheal connector (proximal 1 mm diameter air escape hole) and a plastic mouthpiece (2 cm inner diameter)^12^. Three assessments were made, and the highest value was considered as the end result.

After a 15-minute rest period, the 6MWT was carried out using the guidelines of the American Thoracic Society^13^.

Children first watched a first demonstration of the tests. The speech therapist asked the children to make maximal efforts.

The data-processing software was the SPSS 16.0 (SPSS Inc., Chicago, IL, USA). Student's t test was applied in the analysis of the distance covered in the 6MWT. The Mann-Whitney test was applied in the analysis of the MIP and MEP. The chi-square test was applied to evaluate any association among qualitative variables. The prevalence odds ratio and the 95% confidence interval were measured using the Epi-Info version 6.04d software (Center for Disease Control & Prevention, USA). The significance level was 5%.

The institutional review board of the Medical School - UNICAMP approved this study (no. 849/2008). Before the study, parents or caretakers signed a free informed consent form.

## RESULTS

The sample comprised 107 children; 45 (42.0%) were mouth breathing children, and 62 (58.0%) were nose breathing children. The mean ages were respectively 9.8±0.9 and 9.6±0.9 years (*p*=0.365) (Figure 1). In the mouth breathing group, 29 children (64.5%) were male, and 16 children (35.5%) were female; in the nose breathing group, 23 children (37.1%) were male, and 39 children (62.9%) were female (*p*=0.005). There were no ethnic (*p*= 0.807), weight (*p*= 0.281), height (*p*= 0.958), or body mass index differences (*p*= 0.157) between the two groups.

An altered general posture was observed in 18 mouth breathing children (40.0%) and in 33 nose breathing children (53.2%) (prevalence odds ratio=0.59 [95%CI:0.25-1.37], *p*=0.176). Table 1 shows these data.

**Table 1:** Altered general posture in the groups: mouth breathers (OB) and nasal breathers (NB).

	General posture			POR	
	Altered	Normal	Total	[95% CI]	*p*
OB	18 (40.0%)	27 (60.0%)	45	0.59	
NB	33 (53.2%)	29 (46.8%)	62	[0.25 - 1.37]	

POR - Prevalence odds ratio; CI 95% - 95% confidence interval; *p*= chi-square test probability.

An altered neck posture was observed in 36 mouth breathing children (80.0%) and 30 nose breathing children (48.4%) (prevalence odds ratio=4.27 [95%CI:1.63-11.42], *p*<0.001) (Table 2).

**Table 2:** Altered neck posture in the groups: mouth breathers (OB) and nasal breathers (NB).

	Neck posture			POR	
	Altered	Normal	Total	[95% CI]	*p*
OB	36 (80.0%)	9 (20.0%)	45	4.27	
NB	30 (48.4%)	32 (51.6%)	62	[1.63 - 11.42]	<0.001

POR - Prevalence odds ratio; CI 95% - 95% confidence interval; *p*= chi-square test probability.

The mean MIP was lower in the mouth breathing group (45.0±19.6 × 62.0±22.7; *p*<0.001); similarly, the mean MEP was lower in the mouth breathing group (47.3±17.2 × 58.8±22.3; *p*=0.008). There were no differences between groups in the walked distance in the 6MWT (*p*=0.576) (Table 3).

**Table 3:** Distribution of mean MIP, MEP, and 6MWT results in relation to the breathing pattern, and general and neck posture.

		MIP	MEP	6MWT
	N	Mean±DP	Mean±DP	Mean±DP
Breathing pattern				
OB	45	45.0±19.6	47.3±17.2	624.5±49.4
NB	62	62.0±22.7	58.8±22.3	629.8±47.6
*p*		<0.001	0.008	0.576
General posture				
altered	51	59.1±23.1	58.9±22.0	626.1±41.4
normal	56	51.0±22.3	49.5±19.2	628.9±53.9
*p*		0.070	0.016	0.763
Neck posture				
altered	66	56.3±23.7	56.1±22.2	619.8±49.8
normal	41	52.6±21.8	50.5±18.7	640.0±43.2
*p*		0.360	0.228	0.064

MIP - maximal inspiratory pressure (cm H2O); MEP - maximal expiratory pressure (cm H2O); 6MWT - distance covered (m) in the 6-minute walk test; SD - standard deviation; OB - mouth breather; NB - nose breather.

Table 3 also shows that the mean MEP was highest in the group that had an altered general posture compared to the group with a normal posture (58.9±22.0 × 49.5±19.2; *p*=0.016). There were no differences in the mean MIP (*p*=0.070) and walked distances in the 6MWT (*p*=0.763). There were no differences in the means between the groups with normal and altered neck posture.

There were no differences in the mean MIP, MEP, and walked distance in the 6MWT relative to the presence of altered general posture in the mouth breathing group. On the other hand, subjects with altered general posture had a higher mean MIP in the nose breathing group (67.4-20.4 × 55.9-24.0; *p*=0.048). Table 4 shows these data.

**Table 4:** Distribution of mean MIP, MEP, and 6MWT results in relation to general and neck posture in OB and NB groups.

			MIP	MEP	6MWT
		N	Mean±SD	Mean±SD	Mean±SD
	General posture				
	Altered	18	43.9±20.2	50.0±18.9	629.1±38.7
OB	Normal	27	45.7±19.5	45.6±16.1	621.4±55.9
	*p*		0.700	0.375	0.610
	Altered	33	67.4±20.4	63.8±22.3	624.4±43.4
NB	Normal	29	55.9±24.0	53.1±21.3	625.9±52.0
	*p*		0.048	0.055	0.345
	Neck posture				
	Altered	36	44.1±20.1	46.5±17.4	620.4±51.5
OB	Normal	9	48.3±17.6	50.5±16.6	640.5±37.3
	*p*		0.586	0.548	0.281
	Altered	30	70.8±19.1	67.6±22.0	619.0±48.3
NB	Normal	32	53.7±22.9	50.5±19.4	639.8±45.2
	*p*		0.003	0.004	0.085
	Neck posture				
	OB - altered	36	44.1±20.1	46.5±17.4	620.4±51.5
	NB - altered	30	70.8±19.1	67.6±22.0	619.0±48.3
	*p*		<0.001	<0.001	0.959

MIP - maximal inspiratory pressure (cm H_2_O); MEP - maximal expiratory pressure (cm H_2_O); 6MWT - distance covered (m) in the 6-minute walk test; SD - standard deviation; OB - mouth breather; NB - nose breather

There were no differences in the mean MIP, MEP, and walked distance in the 6MWT among mouth breathing subjects with normal and altered neck posture. The mean MIP (70.8±19.1 × 53.7±22.9; *p*=0.003) and MEP (67.6±22.0 × 50.5±19.4; *p*=0.004) in the nose breathing group were higher in subjects with altered posture (Table 4).

Table 4 shows the distribution of the MIP, MEP, and 6MWT relative to the type of breathing in subjects with altered neck posture. A lower mean MIP (44.2±20.2 × 70.8±19.1; *p*<0.001) and MEP (46.5±17.5 × 67.7±22.1; *p*<0.001) were found in the mouth breathing group. The groups did not differ in the 6MWT (*p*=0.959).

## DISCUSSION

Submaximal exercise capacity, body posture, and respiratory muscle strength (FMR) in children with the mouth breathing syndrome were assessed in this study. No published paper including all of these variables was found in the literature to this date.

Our results showed a higher prevalence of mouth breathing in male subjects; this has also been noted by other authors^2^,^14^. The airways are narrower and the incidence of allergic rhinitis is higher in male children. Allergic rhinitis is considered as one of the main causes of the mouth breathing syndrome^15^.

McEvoy & Grimmer^16^ have pointed out that the posture of children changes from 7 to 12 years as the child adapts to his or her new bodily proportions. Penha et al.^17^ analyzed the body posture of healthy schoolchildren aged from 7 to 10 years and found a high rate of altered postures. Posture compensation takes place at these ages until the body is fully developed. As our sample consisted of children in a similar age group as Penha et al.'s sample, an absence of significant differences in general posture between both groups may be due to postural changes throughout the sample.

The New York test was used to assess general body posture, and specifically the head posture. An anteriorized position of the head is a combination of flexion of the lower portion of the cervical spine and extension of the upper cervical spine; This is the first compensation in posture that mouth breather adopt^4^,^18^. It is thought that this postural change - that starts in the head - gives rise to a cascade of changes in other bodily structures^19^, ^20^, ^21^.

A few studies have assessed body posture in mouth breathing subjects; it is a consensus that anterior tilting of the head is the main change^19^, ^20^, ^21^, ^22^, ^23^, ^24^. This is similar to our finding that mouth breathing was associated with an altered neck posture - the probability of having an anteriorized head is four times higher in mouth breathers.

Analysis of respiratory mechanics (maximal respiratory pressures) showed lower means in the mouth breathing group, compared to nose breathers.

The mouth breathing syndrome includes an altered respiratory biomechanics because of an anteriorized head and ineffective contraction of diaphragmatic and abdominal muscles. Furthermore, mouth breathing requires less muscle effort, which together with inhibition of afferent nasal nerves, results in poorer use of respiratory muscles and progressive muscle weakening^6^,^25^.

A study of the MIP in 37 children with enlarged tonsils showed that the mean MIP was lower, compared to a control (nose breathing) group; our study concurs with this finding^25^. On the other hand, the mean values in Pires et al.'s^26^ study were lower compared to our values (14.60±7.33 × 45.0±19.5 cmH_2_O in the mouth breathing group and 27.58±4.7 × 62.0±22.7 cmH_2_O in the nose breathing group). These results may have been influenced by issues such as: a small sample, age and sex differences, variations in stimulus patterns, different levels of motivation, and subjects recruited from dissimilar settings (community x hospital).

On respiratory muscle strength in mouth breathers, a study of the thoracic perimeter in mouth breathing children found lower values compared to nose breathers^26^. This is probably due to decreased chest expansion, which compromises respiratory muscle activity. Yi et al.'s^23^ finding that diaphragmatic excursion is decreased also underlines that ventilatory mechanics are altered in the mouth breathing syndrome, as we also found.

There were no differences in the mean MIP and MEP in mouth breathing children with different general and neck postures. On the other hand, a worse head posture increased MIP and MEP values in nose breathing children. Another difference in nose breathers was that children with altered general posture had higher MIP values, which suggests that this group of children used these postural changes to compensate and attain higher MIP and MEP values compared to children with normal general and neck posture. Mouth breathing children appear to have a more compromised posture that does not allow any compensating maneuver.

A few authors have evaluated cardiorespiratory function in mouth breathing subjects^27^, ^28^, ^29^, ^30^. Ribeiro & Soares^27^ found lower than predicted spirometry test values (forced expiratory flow 25%-75%, and maximum voluntary ventilation), characterizing a mostly mild to moderate obstructive type ventilation disorder in mouth breathers. The bronchi may also be compromised; increased nasal resistance changes intrathoracic pressure and decreases the pulmonary volume.

Melissant et al.^30^ induced upper airway obstruction during exercise and found that the minute ventilation and elimination of CO_2_ were decreased. These subjects also had hypoventilation, hypoxia, and hypercapnia.

Although mouth breathing may affect exercise capacity, the walked distance in the 6MWT was similar in the mouth and nose breathing groups.

There were anthropometric differences (age, sex, height, weight, and body mass index) in the samples of other studies that used the 6MWT in children, as well as subject recruitment in hospital settings, different 6MWT methods, and assessment of subjects with different diseases, all of which made comparisons with our results difficult^31^,^32^. It should be noted that different approaches in the method and description of walk tests affect how these studies are interpreted, and make any comparison among them difficult.

It is known that several factors affect the walked distance in the 6MWT, both negatively (lower height, shorter lower limbs, advanced age, high body weight, female sex, altered cognition, shorter aisles and therefore more turns, and chronic respiratory, cardiovascular, or orthopedic diseases) and positively (height, male sex, motivation, training before the test, a few drugs, and oxygen supplementation)^13^.

The mouth breathing group had more male subjects, which may have increased its mean walked distance. Female subjects have lower vital capacity and maximum expiratory flows, and smaller diffusion surface. These differences may have an integrated effect on ventilation, respiratory muscle work, and gas exchanges during exercise. Thus, exercise tolerance is lower in female subjects as a result of more limited expiratory flow and respiratory work^33^. A predominance of males in the mouth breathing group may have overestimated the values in the walked distance (6MWT).

A maximal cardiopulmonary test would have been more sensitive to detect dysfunctions, as the 6MWT is indicated for more limiting diseases; it is, however, easier to carry out.

Analyzing only subjects with altered posture, the study revealed that MIP and MEP values were lower in the mouth breathing group, suggesting that mouth breathing has more influence on respiratory biomechanics than having an altered neck posture.

The study variables showed that mouth breathing affects both posture and respiratory muscle strength; posture and the respiratory system are proportionally involved, but with no effect on exercise tolerance. In the long term, losses in ventilatory mechanics may be reinforced by altered posture, which may have a negative effect on exercise capacity.

Studies with larger samples, higher age groups, children in hospital or clinical settings, use of a maximal cardiopulmonary test, and a longitudinal design may clarify these relationships, which have not been investigated in depth. Given these changes, we note that early interventions on the muscle-skeletal and respiratory systems are important. Specific evaluation methods are needed to clarify the numerous effects of the mouth breathing syndrome.

## CONCLUSION

In the study group, mouth breathing children had a higher rate of altered neck posture and decreased respiratory muscle strength compared to nose breathing children.
